# Supplementary material for: Identification and Functional Analysis of the Regulatory Elements in the pHSPA6 Promoter
Source: Genes (Basel). 2022 Jan 21;13(2):189. doi: 10.3390/genes13020189 (PMC8872561; doi:10.3390/genes13020189)
Supplement: Supplementary file 1 [file genes-13-00189-s001.zip › genes-1521058-supplementary.pdf]

Identification and functional analysis of the regulatory elements in the p*HSPA6* promoter

Shuyu Jiao#[1], Chunyan Bai#[1], Chunyun Qi [1], Heyong Wu[1], Lanxin Hu[1], Feng Li[1], Kang Yang[1], Chuheng Zhao[1], Hongsheng Ouyang[1,2,3,4], Daxin Pang[1,2,3,4], Xiaochun Tang*[2,3,4], Zicong Xie *[1]

1.College of Animal Science, Jilin University, Changchun 130062, China

2.Key Lab for Zoonoses Research, Ministry of Education, Animal Genome Editing Technology Innovation Center, Jilin Province, Jilin University, Changchun 130062, China

3. Chongqing Research Institute, Jilin University, Chongqing 401123, China

4. Chongqing Jitang Biotechnology Research Institute Co., Ltd

* Corresponding auther. Xinzhu Road, Jilin University, Changchun, Jilin, PR China

E-mail addersses: xzc@jlu.edu.cn (ZC. Xie), xiaochuntang@jlu.edu.cn (XC. Tang)

Address: 5333#, Xinzhu Road, Changchun 130062, China; Tel.: (86) 431-87836175; Fax: (86) 431-86758018

The Supplementary Data

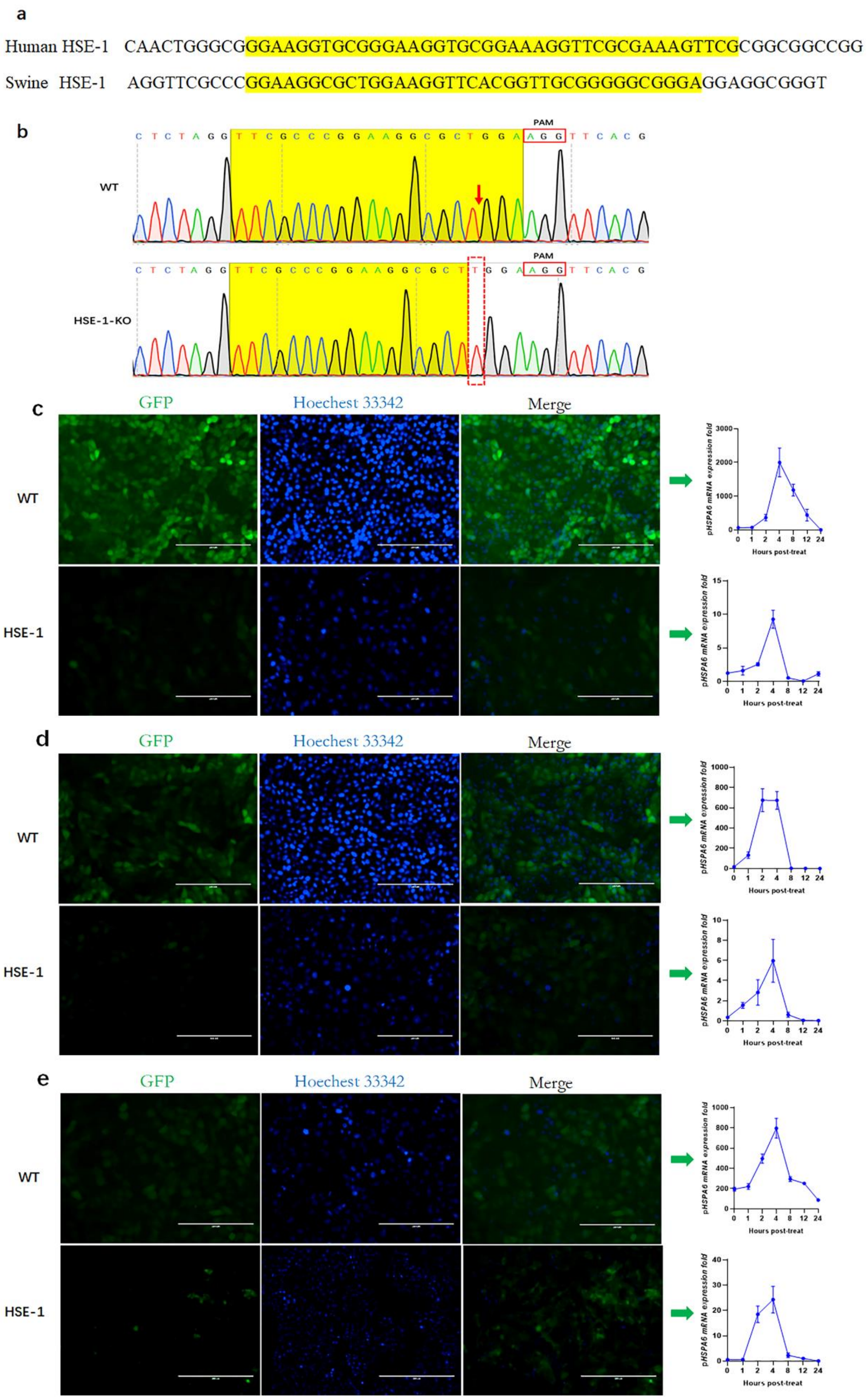

Figure S1. Identification of HSE-1 and its effect on p*HSPA6* expression. (a) swine HSE-1 was identified by alignment with humans. (b) Sanger sequencing analyses were used to further confirm the expression of sgRNA-H1 in positive clone HSE-1. (c) Fluorescence

microscopy and qPCR analysis of the effects of copper sulfate on p*HSPA6*-induced expression in WT and positive clone cells (line chart. n=3. graphs show the mean ± S.E.M.). (d) Fluorescence microscopy and qPCR analysis of the effects of MG132 on p*HSPA6*-induced expression in WT and positive clone cells (line chart. n=3. graphs show the mean ± S.E.M.). (e) Fluorescence microscopy and qPCR analysis of the effects of curcumin on p*HSPA6*-induced expression in WT and positive clone cells (line chart. n=3. graphs show the mean ± S.E.M.). Endogenous GAPDH was used as an RNA quality and loading control.

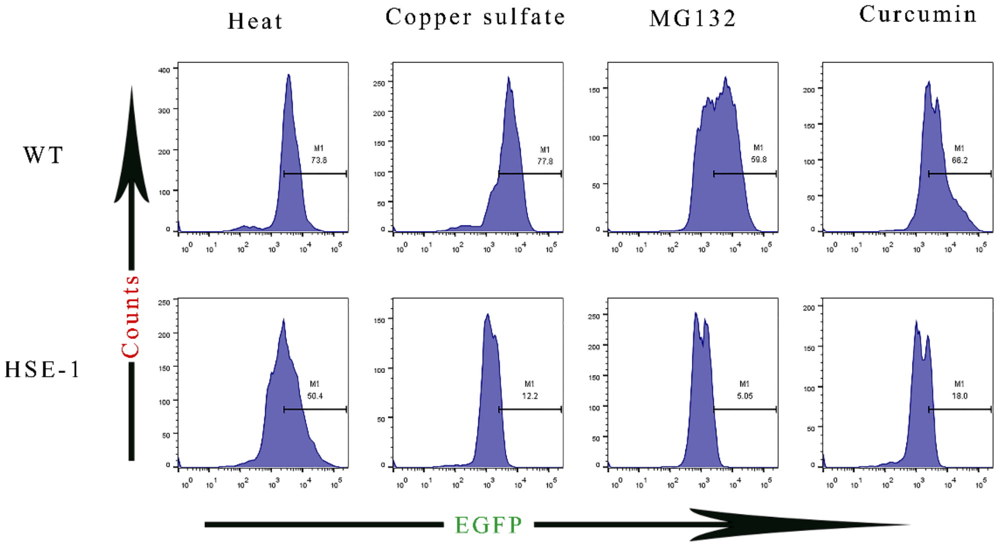

Figure S2. EGFP fluorescence in WT and HSE-1 cells was confirmed via FACS.

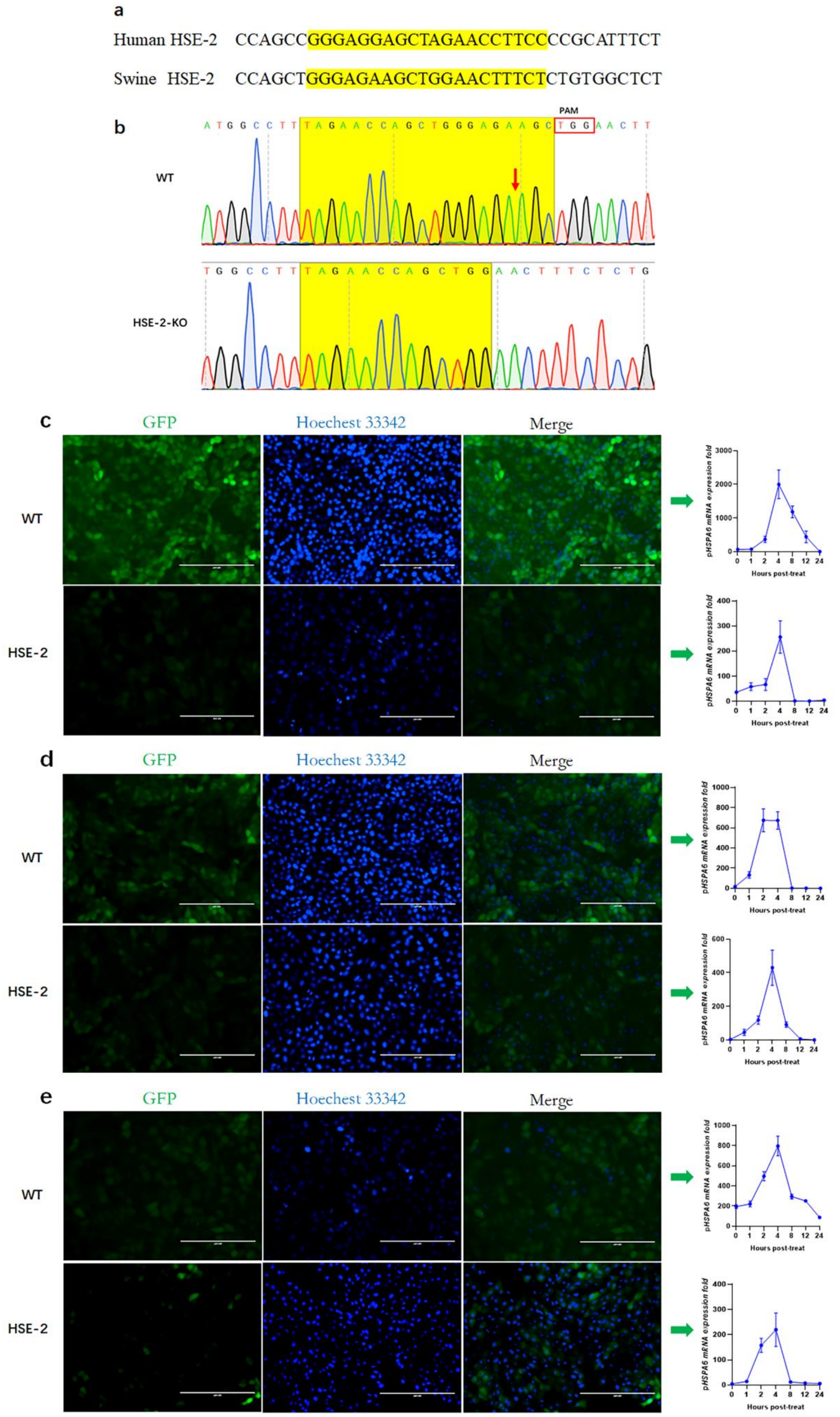

Figure S3. Identification of HSE-2 and its effect on p*HSPA6* expression. (a) swine HSE-2 was identified by alignment with humans. (b) Sanger sequencing analyses were used to further confirm the expression of sgRNA-H2 in positive clone HSE-2. (c) Fluorescence microscopy and qPCR analysis of the effects of copper sulfate on p*HSPA6*-induced

expression in WT and positive clone cells (line chart. n=3. graphs show the mean ± S.E.M.). (d) Fluorescence microscopy and qPCR analysis of the effects of MG132 on p*HSPA6*-induced expression in WT and positive clone cells (line chart. n=3. graphs show the mean ± S.E.M.). (e) Fluorescence microscopy and qPCR analysis of the effects of curcumin on p*HSPA6*-induced expression in WT and positive clone cells (line chart. n=3. graphs show the mean ± S.E.M.). Endogenous GAPDH was used as an RNA quality and loading control.

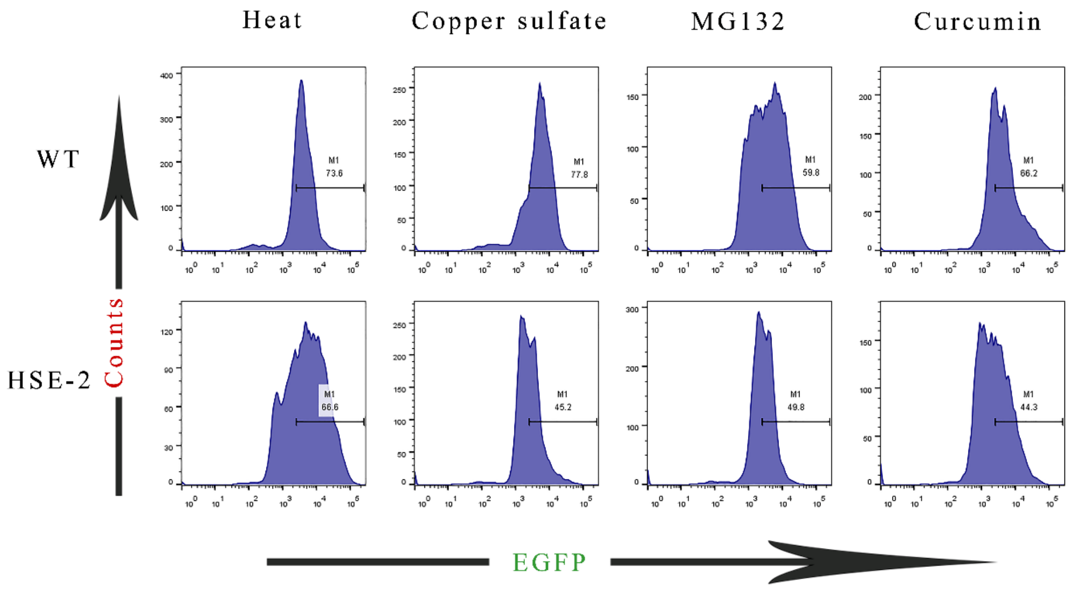

Figure S4. EGFP fluorescence in WT and HSE-2 cells was confirmed via FACS.

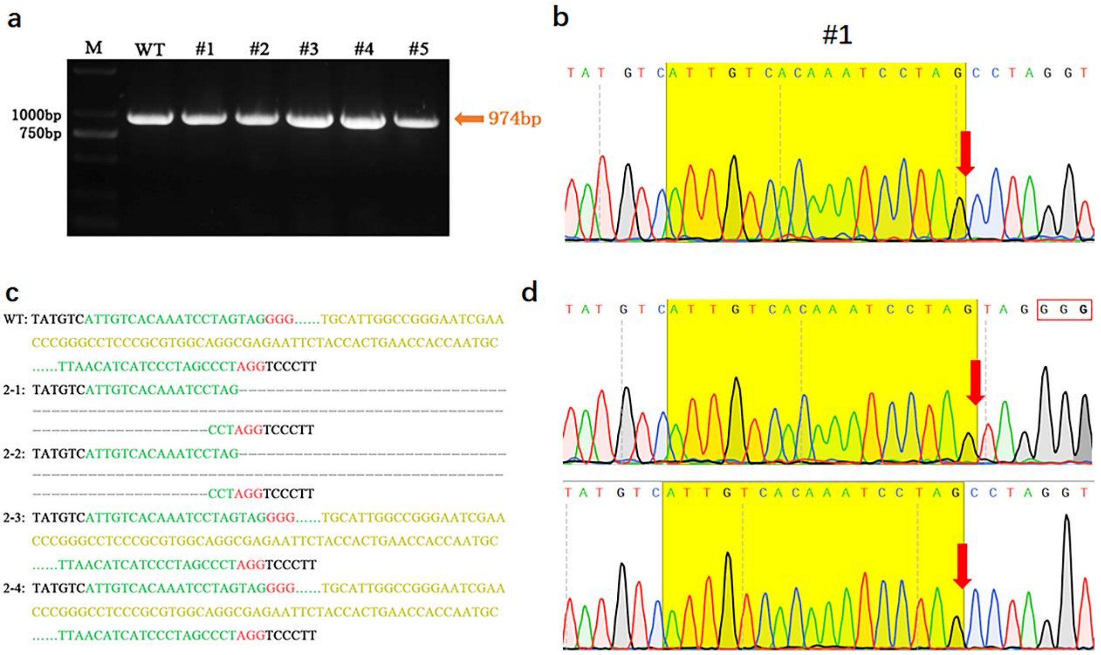

Figure S5. Selection of CR knockout cell clones. (a) Genomic PCR analysis confirmed the knock-in events of the EGFP gene at the p*HSPA6* locus. (b) Sanger sequencing analyses were used to further confirm the expression of sgRNA1-1 and sgRNA1-2 in positive

clone #1; the red arrows indicate the cleavage sites. (c) Diagram of positive clone #2 after pLB vector cloning. (d) Sanger sequencing analyses were used to further confirm the expression of sgRNA1-1 and sgRNA1-2 in positive clone #2; PAMs are marked in red block and the cleavage sites are labelled with red arrows.

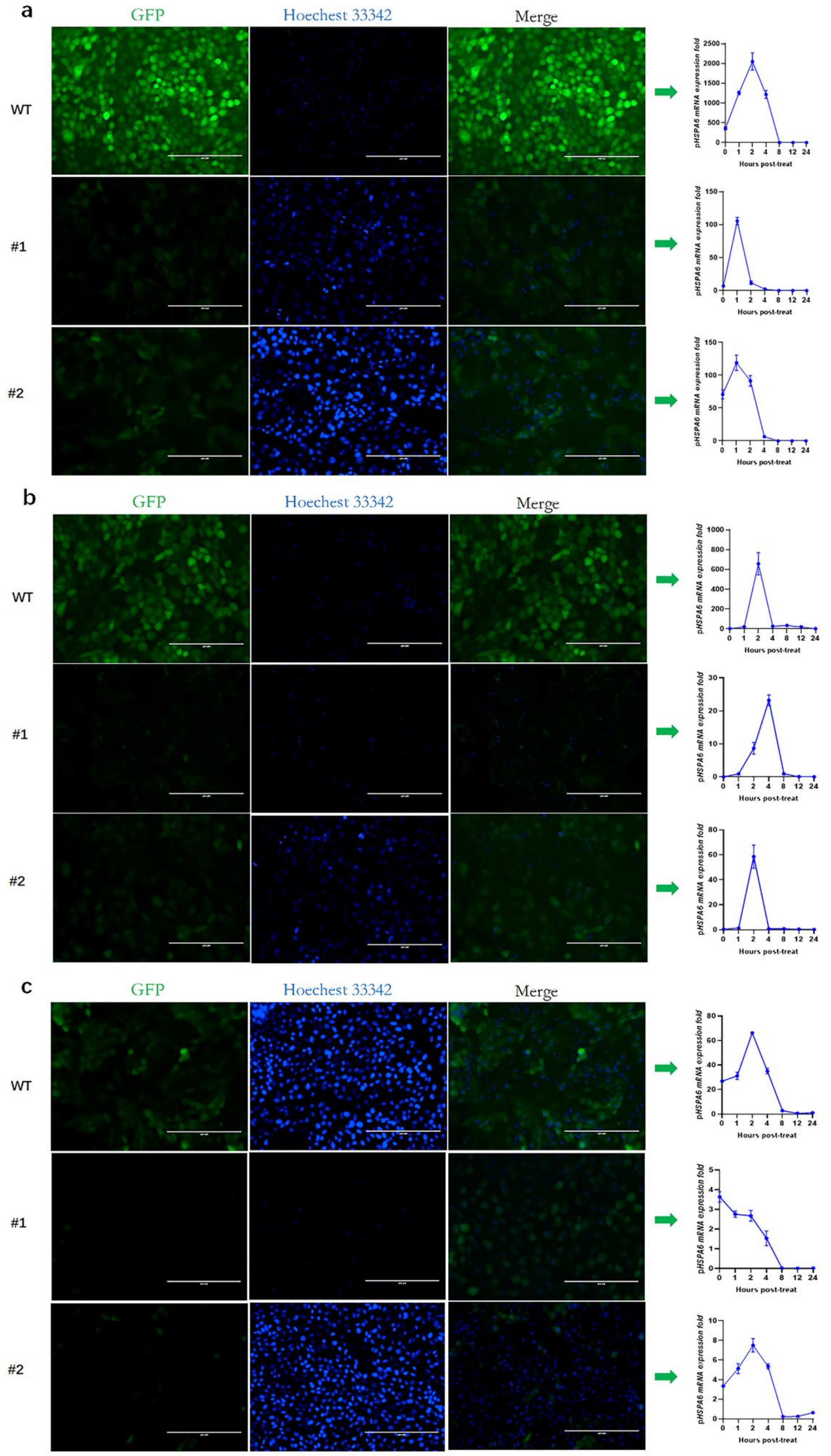

Figure S6. p*HSPA6* expression analysis on CR under stressors. (a) Fluorescence microscopy and qPCR analysis of the effects of copper sulfate on p*HSPA6*-induced

expression in WT and positive clone cells (line chart. n=3. graphs show the mean ± S.E.M.). (b) Fluorescence microscopy and qPCR analysis of the effects of MG132 on p*HSPA6*-induced expression in WT and positive clone cells (line chart. n=3. graphs show the mean ± S.E.M.). (c) Fluorescence microscopy and qPCR analysis of the effects of curcumin on p*HSPA6*-induced expression in WT and positive clone cells (line chart. n=3. graphs show the mean ± S.E.M.). Endogenous GAPDH was used as an RNA quality and loading control.

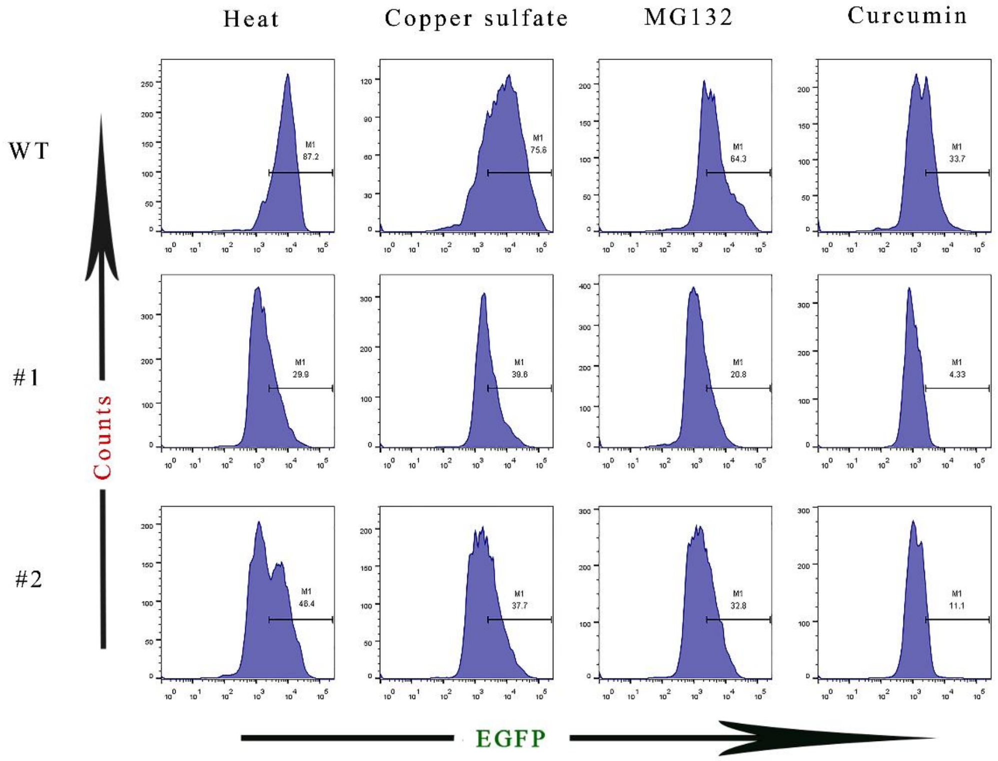

Figure S7. EGFP fluorescence in WT, #1 and #2 cells was confirmed via FACS.
